# A Missense Change in the *ATG4D* Gene Links Aberrant Autophagy to a Neurodegenerative Vacuolar Storage Disease

**DOI:** 10.1371/journal.pgen.1005169

**Published:** 2015-04-15

**Authors:** Kaisa Kyöstilä, Pernilla Syrjä, Vidhya Jagannathan, Gayathri Chandrasekar, Tarja S. Jokinen, Eija H. Seppälä, Doreen Becker, Michaela Drögemüller, Elisabeth Dietschi, Cord Drögemüller, Johann Lang, Frank Steffen, Cecilia Rohdin, Karin H. Jäderlund, Anu K. Lappalainen, Kerstin Hahn, Peter Wohlsein, Wolfgang Baumgärtner, Diana Henke, Anna Oevermann, Juha Kere, Hannes Lohi, Tosso Leeb

**Affiliations:** 1 Department of Veterinary Biosciences, University of Helsinki, Helsinki, Finland; 2 Research Programs Unit, Molecular Neurology, University of Helsinki, Helsinki, Finland; 3 Department of Molecular Genetics, Folkhälsan Institute of Genetics, Helsinki, Finland; 4 Institute of Genetics, Vetsuisse Faculty, University of Bern, Bern, Switzerland; 5 Department of Biosciences and Nutrition, Karolinska Institutet, Huddinge, Sweden; 6 Department of Equine and Small Animal Medicine, University of Helsinki, Helsinki, Finland; 7 Department of Clinical Veterinary Medicine, Division of Clinical Radiology, Vetsuisse Faculty, University of Bern, Bern, Switzerland; 8 Neurology Service, Department of Small Animals, Vetsuisse Faculty, University of Zurich, Zurich, Switzerland; 9 University Animal Hospital, Swedish University of Agricultural Sciences, Uppsala, Sweden; 10 Department of Companion Animal Clinical Sciences, Norwegian University of Life Sciences, Oslo, Norway; 11 Department of Pathology, University of Veterinary Medicine Hannover, Hannover, Germany; 12 Division of Neurological Sciences, Vetsuisse Faculty, University of Bern, Bern, Switzerland; 13 Science for Life Laboratory, Karolinska Institutet, Solna, Sweden; Stanford University School of Medicine, United States of America

## Abstract

Inherited neurodegenerative disorders are debilitating diseases that occur across different species. We have performed clinical, pathological and genetic studies to characterize a novel canine neurodegenerative disease present in the Lagotto Romagnolo dog breed. Affected dogs suffer from progressive cerebellar ataxia, sometimes accompanied by episodic nystagmus and behavioral changes. Histological examination revealed unique pathological changes, including profound neuronal cytoplasmic vacuolization in the nervous system, as well as spheroid formation and cytoplasmic aggregation of vacuoles in secretory epithelial tissues and mesenchymal cells. Genetic analyses uncovered a missense change, c.1288G>A; p.A430T, in the autophagy-related *ATG4D* gene on canine chromosome 20 with a highly significant disease association (p = 3.8 x 10^-136^) in a cohort of more than 2300 Lagotto Romagnolo dogs. *ATG4D* encodes a poorly characterized cysteine protease belonging to the macroautophagy pathway. Accordingly, our histological analyses indicated altered autophagic flux in affected tissues. The knockdown of the zebrafish homologue *atg4da* resulted in a widespread developmental disturbance and neurodegeneration in the central nervous system. Our study describes a previously unknown canine neurological disease with particular pathological features and implicates the ATG4D protein as an important autophagy mediator in neuronal homeostasis. The canine phenotype serves as a model to delineate the disease-causing pathological mechanism(s) and ATG4D function, and can also be used to explore treatment options. Furthermore, our results reveal a novel candidate gene for human neurodegeneration and enable the development of a genetic test for veterinary diagnostic and breeding purposes.

## Author Summary

Neurodegenerative disorders affect millions of people worldwide. We describe a novel neurodegenerative disease in a canine model, characterized by progressive cerebellar ataxia and cellular vacuolization. Our genetic analyses identified a single nucleotide change in the autophagy-related *ATG4D* gene in affected dogs. The *ATG4D* gene has not been linked to inherited diseases before. The autophagy-lysosome pathway plays an important role in degrading and recycling different cellular components. Disturbed autophagy has been reported in several different diseases but mutations in core autophagy components are rare. Histological analyses of affected canine brain tissues revealed altered autophagic flux, and a knockdown of the gene in the zebrafish model caused marked neurodevelopmental alterations and neurodegeneration. Our findings identify a new disease-causing pathway and implicate the ATG4D protease as an important mediator for neuronal homeostasis. Furthermore, our study establishes a large animal model to investigate the role of ATG4D in autophagy and to test possible treatment options.

## Introduction

The intracellular homeostasis of neurons, especially in the cerebellar Purkinje cells, is easily disturbed by dysfunction in degradative processes and accumulation of different cellular materials [1]. The autophagy-lysosome pathway [2] and the ubiquitin-proteasome system [3] are two major cellular degradation pathways. The autophagy (or self-eating) process is particularly important in the degradation of organelles and long-lived proteins, whereas the proteasome complex targets more short-lived proteins [4, 5]. Macroautophagy (usually referred to simply as autophagy) is an evolutionary conserved intracellular process, in which proteins and organelles are sequestered within double-membrane autophagosomes and delivered to the lysosome for degradation. This recycling process is orchestrated by several different autophagy related (ATG) proteins in order to maintain proper cellular homeostasis under both basal state and stressful conditions, such as nutrient deprivation [2]. The ubiquitin-proteasome system and the autophagy-lysosome pathway are interlinked [4], and their dysfunction has been implicated in various detrimental neurodegenerative disorders, such as inherited ataxias, Alzheimer and Parkinson disease, and the lysosomal storage disorders (LSDs) [6, 7].

The LSDs form a family of around 50 inherited metabolic diseases characterized by accumulation of macromolecules within intracellular vacuoles of the endosomal-autophagic-lysosomal pathways [8, 9]. LSDs can be subgrouped on the basis of the stored material, ranging from carbohydrates (e.g. mucopolysaccharidoses) to different types of lipids (e.g. sphingolipidoses) and proteins, or a combination of these [9]. Although the disease usually involves multiple organs, central nervous system (CNS) dysfunction and neurodegeneration are present in the majority of LSDs [8–10]. The classical causative mutations disrupt lysosomal enzymes, leading to accumulation of their unprocessed substrates within lysosomes [8, 10]. However, there are LSDs that differ from this classical example. Dysfunction of other types of proteins important for lysosomal function, such as the lysosomal membrane protein *LAMP2* [11], can also cause LSD. Furthermore, the pathological changes in some LSDs appear to result rather from defects in intracellular membrane trafficking than in processing of the lysosomal substrates, and many show signs of altered autophagic flow [12]. In fact, the involvement of the autophagy pathways in several LSDs has prompted a suggestion that LSDs could in part be seen as autophagy disorders [13].

Inherited neurological diseases also occur in the domestic dog (*Canis lupus familiaris*), and many are caused by mutations in the same genes as corresponding human conditions [14]. In the present work, we report a novel inherited neurodegenerative condition in the Lagotto Romagnolo (LR) dog breed, and describe its clinical and pathological characteristics and the likely genetic cause. The affected dogs present with progressive cerebellar ataxia, and histological findings reveal intracellular vacuolization, altered neural autophagy and neurodegeneration. Our results suggest that the disorder is caused by a recessive missense mutation in the *ATG4D* gene, which encodes an autophagy-related proteinase [15].

## Results

### Clinical characterization reveals progressive ataxia with occasional nystagmus and behavioral abnormalities

A particular neurodegenerative disease was first recognized in three LRs that presented with progressive neurological signs. The three affected dogs comprised two full siblings and a distantly related dog. Similar clinical history, clinical examination findings and corresponding histological changes in post mortem pathological examination in all three dogs indicated a shared disease etiology.

During the course of the genetic study, we performed a detailed neurological examination in altogether 16 affected LR dogs, and another six LRs were reported by their owners to suffer from comparable neurological signs. The typical clinical presentation in affected dogs was progressive ataxia (S1 Video), and many of the dog owners reported that their dogs had been a bit clumsy even before they noticed obvious ataxia. Ten of the 22 affected dogs had episodes of abnormal eye movements (nystagmus) and this was the first clinical sign noticed by the owners of seven affected dogs. Later in the course of the disease, the owners of seven affected LRs reported behavioral changes, such as restlessness, depression and aggression towards people or other dogs. The age at onset of clinical signs varied considerably between the 22 dogs; the first clinical signs were noticed at the mean age of 23 months, ranging from 4 months to 4 years. The rate of progression of clinical signs to a point where euthanasia had to be considered also varied from months to years.

The neurological examination in 16 affected LRs revealed a mild to severe cerebellar ataxia in all examined dogs. The majority of dogs had normal paw positioning responses when postural reactions were tested but showed delayed onset of correction in hopping reactions. Spinal reflexes were normal except for decreased or absent patellar reflexes in five dogs. Menace reaction was decreased in eight dogs, and exaggerated in one dog. Positional nystagmus was visible in four dogs during the neurological examination. Magnetic resonance imaging of the brain was performed in 11 affected dogs. The principal findings included signs of mild atrophy of the cerebellum in nine dogs and of the forebrain in six dogs. In five dogs, lateral ventricles were enlarged. A small corpus callosum was detected in three affected dogs when compared to age matched LRs. In two affected dogs, the brain imaging was unremarkable.

### Pathological findings indicate disturbed autophagy and vesicular trafficking

**Fig 1:**
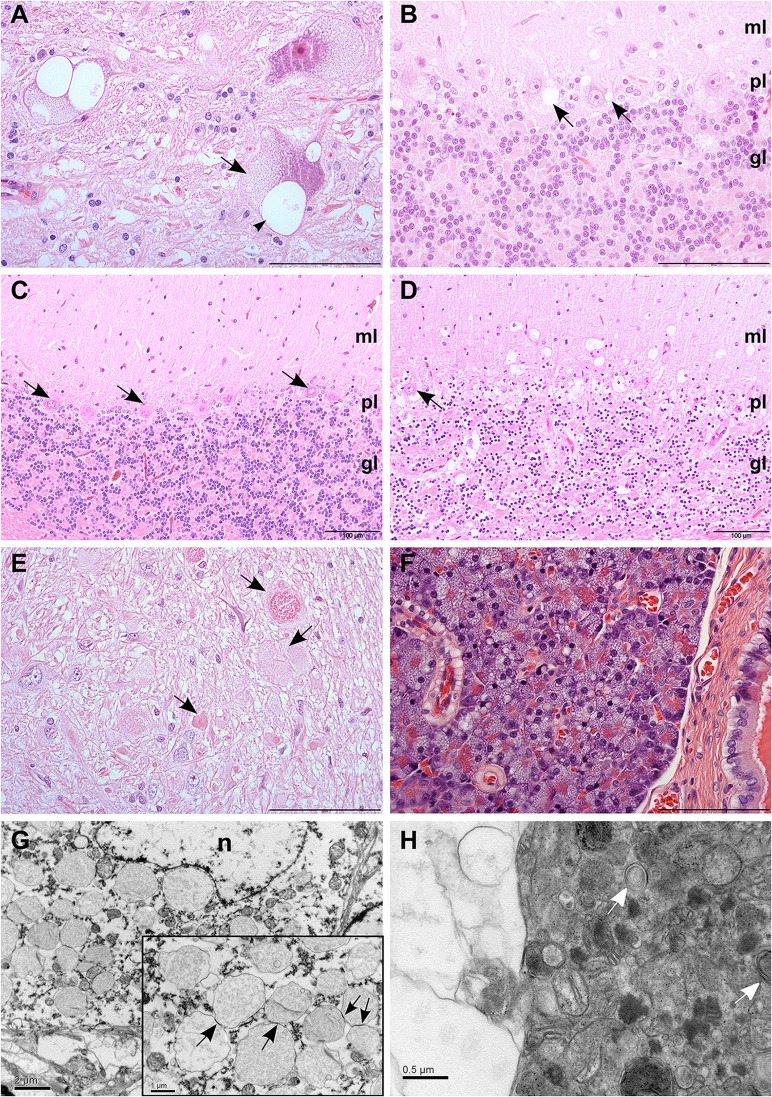
Histological findings in neurons and pancreas. (A) Swelling of neurons in the vestibular nucleus due to fine vesiculation (arrows) and clear vacuolization (arrowhead) of the cytoplasm. HE, scale bar 100 μm. (B) Clear cytoplasmic vacuolization (arrows) in cerebellar cortical Purkinje cells. HE, scale bar 100 μm. (C) Normal cerebellar cortex of an unaffected dog shows viable Purkinje cells (arrows) and a dense granular cell layer. HE, scale bar 100 μm. (D) Marked neuronal loss is present the cerebellar cortex of an affected dog. The number of neurons in the granular cell layer is reduced and only scattered Purkinje cells remain (arrow). HE, scale bar 100 μm. (E) Axonal spheroids of varying quality were seen in the white matter (arrows) of cerebellum and brainstem. HE, scale bar 100 μm. (F) Diffuse cytoplasmic vacuolization of the exocrine pancreatic acinar cells. HE, scale bar 100 μm. (G) Purkinje cell with numerous single-membrane bound, cytoplasmic vacuoles tethering to each other (arrows, inset). Electronmicrograph, scale bar 2 μm. Inset: scale bar 1 μm. (H) Axonal spheroid containing aggregated degenerated mitochondria, occasional double-membrane-bound autophagosomes (arrows) and free electron dense material, compressed by a peripheral clear vacuolar space. Electronmicrograph, scale bar 0.5 μm. Abbreviations: n, nucleus; ml, molecular layer; pl, Purkinje cell layer; gl, granular cell layer.

We performed pathological examination on seven LRs that were euthanized due to progressive neurological signs. Atrophy of the cerebellum was clearly visible on macroscopic examination in two of the examined dogs. Histological examination revealed widespread swelling and clear vacuolization of the neuronal cytoplasm, diffusely affecting the central and peripheral nervous system. The cytoplasmic vacuolization varied from fine vesiculation to large confluent vacuoles (Fig 1A). The cerebellar cortex was consistently affected (Fig 1B), showing marked progressive Purkinje cell loss and granular cell depletion, especially in dogs with a prolonged clinical course or more severe clinical signs (Fig 1C and 1D) The deep cerebellar nuclei, nucleus ruber, nucleus vestibularis and the lateral and medial geniculate nuclei also showed consistent, severe changes. The lesions were milder in the cerebral cortex, the basal ganglia and in specific nuclei, such as the oculomotor and hypoglossal nucleus. Disturbed axonal transport was evident as numerous morphologically diverse axonal spheroids (Fig 1E) in the cerebellar white matter, in the thalamic, brainstem and cerebellar nuclei as well as in the dorsal funiculus of the spinal cord. The spheroids were accompanied by mild to moderate astrocytosis of the cerebellar and brainstem white matter, indicated by an increase in glial fibrillary acidic protein (GFAP)-positive cells.

In addition to the findings in neural tissue, we detected hypertrophy and vesicular vacuolization of the cytoplasm in several secretory epithelial cells, including the cells of the choroid plexus and the subcommisural organ. Outside of the nervous system, vacuolization affected pancreatic acinar cells (Fig 1F), the parathyroid gland, adrenal cortical cells, the prostate, the salivary glands, the mammary gland and bronchial epithelial cells. Furthermore, vacuolization and granular aggregates were present in cells of mesodermal origin, as seen in smooth muscle cells, in the vascular tunica media and occasionally in endothelial cells, but also in cells with macrophage morphology in lymphoid tissue, pulmonary alveoli and in GFAP-negative glial cells scattered along the interface of Purkinje and granular cell layers and throughout the cerebellar white matter. Furthermore, fine vesicular vacuolization of the cytoplasm was seen in the apocrine sweat glands in skin biopsies of three live dogs that suffered from corresponding clinical signs. This vacuolization was comparable to that present in the secretory epithelia of the necropsied dogs.

The content of the neuronal vacuoles did not stain in hematoxylin-eosin (HE) staining and was periodic-acid-Schiff´s (PAS) negative, which suggests that glycogen, glycoprotein, glycolipid or lipofuscin did not accumulate within the vacuoles. Electron microscopy sections of Purkinje cells showed single membrane bound cytoplasmic vacuoles of varying size that either appeared empty or contained very few small membranous profiles or lucent floccular material (Fig 1G). The vacuoles tethered and formed contact sites reminiscent of hemifusion (Fig 1G inset). The axonal swellings contained peripherally coalescing clear vacuoles that compressed degenerated mitochondria, occasional double-membrane-bound autophagosomes, and free electron dense aggregated material (Fig 1H).

### Genetic analyses reveal a missense variant in the *ATG4D* gene

**Fig 2:**
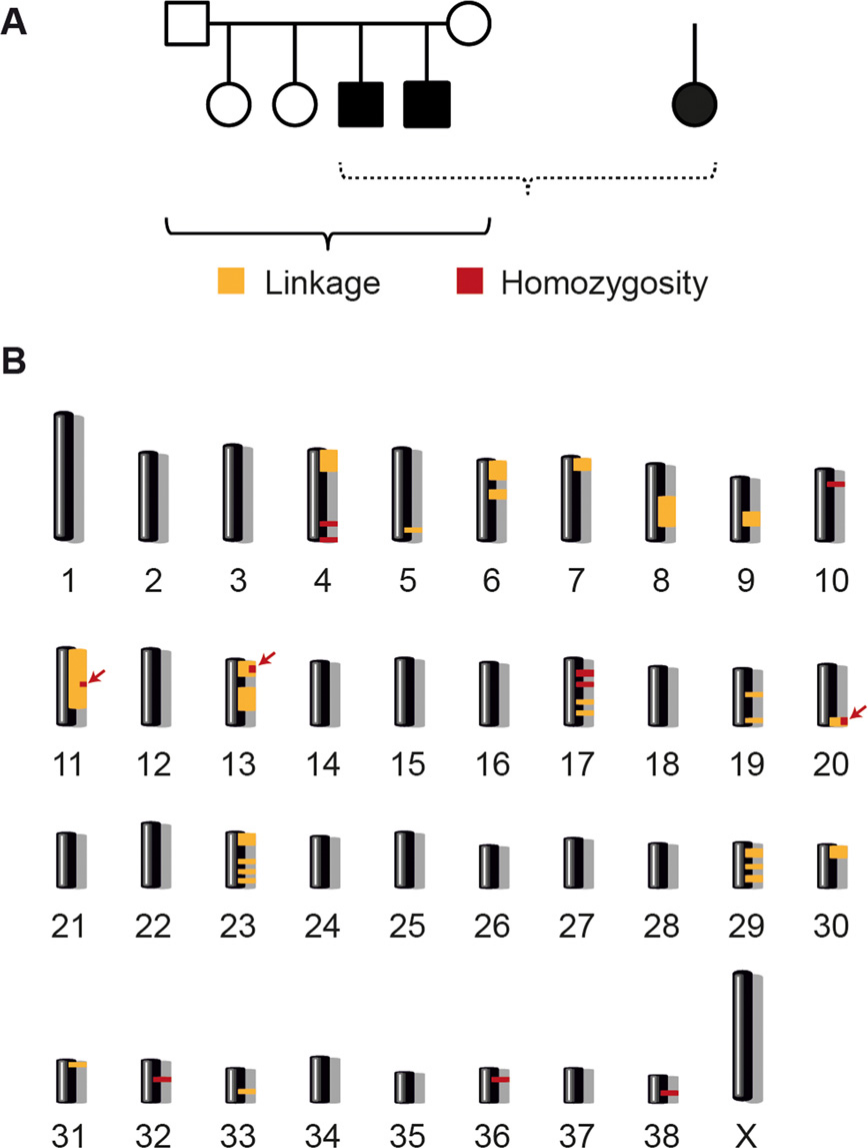
Linkage analysis and homozygosity mapping. (A) A family comprising six animals and one very distantly related case were available for the initial mapping of the disease locus. We performed parametric linkage analysis for a recessive trait in the family and homozygosity analysis across the three cases. (B) The analyses yielded 25 linked genome segments (orange) and 11 homozygous genome segments (red). Only three regions on chromosomes 11, 13, and 20 showed both linkage and homozygosity and were considered the critical intervals (arrows).

At the time the genetic study was initiated, DNA samples had been obtained from only three affected dogs. Two of these were littermates from a Finnish LR family, of which we also had samples from two non-affected full siblings and both parents (Fig 2A). The third affected dog was an isolated case from Switzerland. The phenotype in all three cases had been confirmed by post mortem pathological examination. These three cases and the four controls were genotyped on Illumina canine arrays containing more than 170 k SNPs.

We analyzed the SNP array data by performing linkage analysis and homozygosity mapping. Parametric linkage analysis was carried out for the Finnish LR family under a fully penetrant, monogenic, autosomal recessive model of inheritance. Positive LOD scores were obtained for altogether 25 genome segments that contained 276 Mb of sequence (S1 Table). The three available cases were then analyzed to identify extended regions of homozygosity with simultaneous allele sharing. Based on the pedigree records, we hypothesized that all affected dogs most likely were inbred to one single founder animal. Under this scenario, the affected individuals were expected to be identical by descent (IBD) for the causative mutation and flanking chromosomal segments. The homozygosity mapping identified 11 genome regions that fulfilled our homozygosity search criteria, with a total size of 38Mb (S2 Table). The linked intervals from the family of six were intersected with the homozygous intervals from the three cases. Only three chromosomal segments, on canine chromosomes 11, 13 and 20, were found to be overlapping. These three segments had a combined size of 19 Mb, and were considered the minimal critical interval for the subsequent analyses (Fig 2B and S3 Table).

In order to obtain a comprehensive overview of all variants in the 19 Mb critical interval, we sequenced the whole genome of one pathologically confirmed affected LR. Nearly 210 million 2 x 100 bp paired-end reads were collected from a shotgun fragment library, corresponding to 15.5x coverage of the genome. Single nucleotide and indel variants were called with respect to the reference genome. Across the entire genome, ~7.3 million variants were detected, of which ~2.9 million were homozygous (Table 1). Within the critical intervals, there were 31,016 variants, of which 220 were predicted to be non-synonymous. The variants in the affected LR were filtered against the genomes of 118 dogs from various different breeds that had been sequenced for other ongoing studies (S4 Table). We hypothesized that the causative variant should be completely absent in the other breeds. After this filtering step, only five private homozygous variants remained within the critical intervals (Table 2). One of these variants was intergenic and three were intronic. The remaining fifth variant was the only non-synonymous variant (Chr20:50,618,958C>T). It represented a missense change, c.1288G>A; p.A430T, in the autophagy related 4D, cysteine peptidase gene (*ATG4D*).

**Table 1:** Variants detected by whole genome re-sequencing of an affected Lagotto Romagnolo.

Filtering step	Number of variants
Variants in the whole genome^a^	2,944,010
Variants in the critical intervals on CFA 11, 13, and 20	31,016
Variants in the critical intervals that were absent from 118 other dog genomes	5
Non-synonymous variants in the whole genome	9,871
Non-synonymous variants in the critical intervals on CFA 11, 13, and 20	220
Non-synonymous variants in the critical intervals that were absent from 118 other dog genomes	1

^a^The sequences were compared to the reference genome (CanFam 3.1) from a Boxer. Only those variants that were homozygous in the affected Lagotto Romagnolo are reported.

**Table 2:** Private sequence variants in an affected dog in the critical intervals.

Chr	Position (bp)^a^	Ref. allele	Alt. allele	Gene	Variant (transcript)	Variant (protein)	Trancript reference
Chr13	5,167,594	CTGTGTGTA	C	Intergenic^b^	-	-	-
Chr13	5,635,540	A	T	DPYS	c.790+3201T>A	(intron)	XM_003639441.2
Chr20	50,618,958	C	T	ATG4D	c.1288G>A	p.A430T	XM_542069.3
Chr20	50,679,224	C	A	PDE4A	c.1548+118G>T	(intron)	XM_003432782.3
Chr20	53,431,352	G	C	EMR1	c.1366+159C>G	(intron)	XM_005632337.1

^a^Positions refer to CanFam 3, NCBI annotation release 103.

^b^Distances to the 5’- and 3’-flanking genes (LOC102152837 and LOC102154253) are 190,888 and 266,309 bp, respectively.

**Fig 3:**
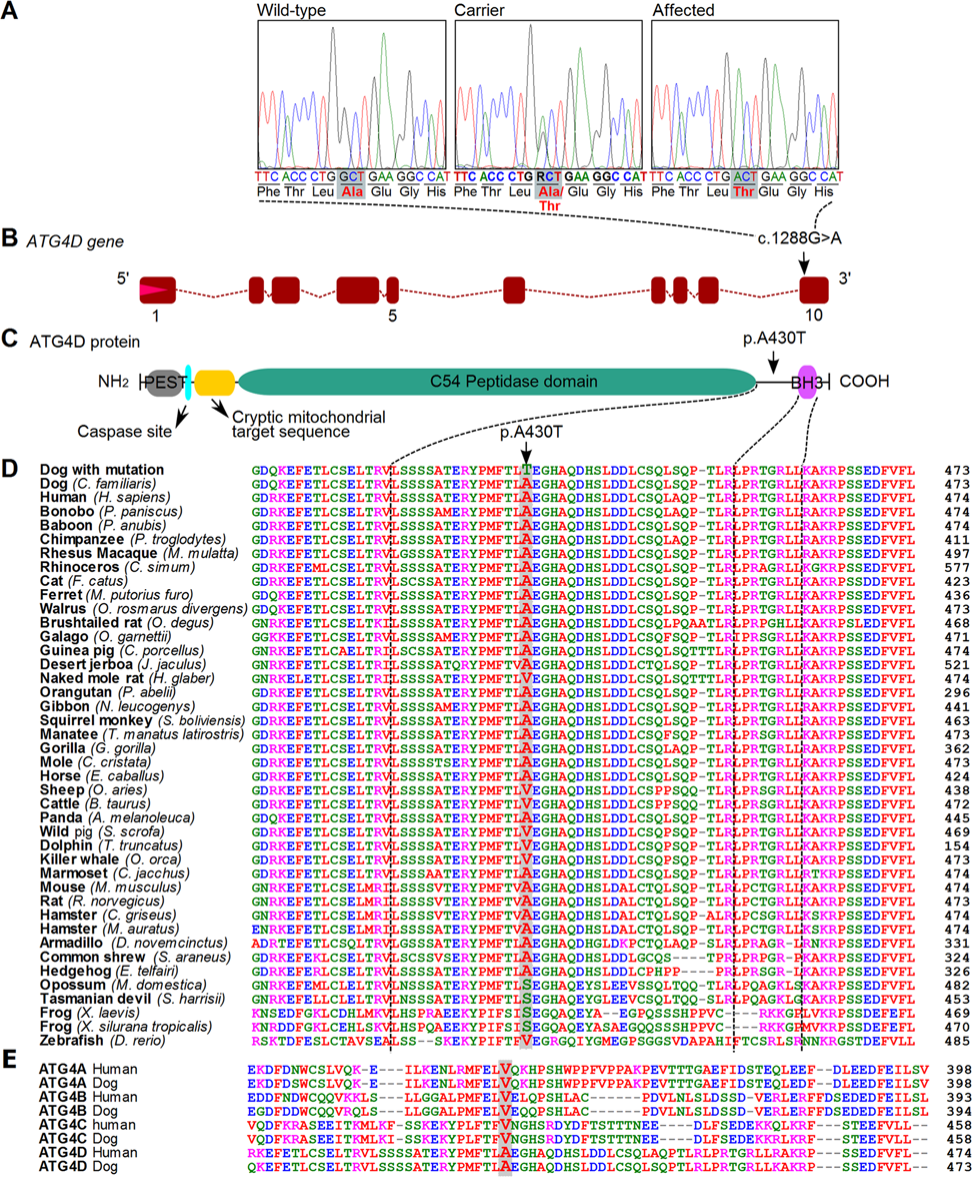
A missense variant in the *ATG4D* gene. (A) Chromatograms showing the c.1288G>A variant in a wild-type, carrier and an affected dog. (B) A schematic presentation of the canine *ATG4D* gene. The missense variant is positioned in the last exon of the gene. (C) The domain structure of the ATG4D protein. The p.A430T change is situated between the functional domains near the carboxy-terminus. (D) The canine ATG4D 430 alanine residue shows a moderate degree of conservation across the animal kingdom. (E) Conservation of the 430 alanine in the ATG4 protein family in human and dog.

The *ATG4D* c.1288G>A variant was then confirmed by Sanger sequencing (Fig 3A) and genotyped in altogether 2,352 LRs. In the entire LR cohort, 25 (1%) dogs were homozygous for the variant allele (A/A), 266 (11%) dogs were heterozygous (G/A) and 2,061 (88%) were homozygous for the reference allele (G/G) (S5 Table). Out of the 25 dogs that were homozygous for the variant, 22 were suffering or had suffered from compatible clinical signs (S6 Table). Seven of these had been confirmed as affected through an autopsy and three through a skin biopsy. Three dogs homozygous for the variant were classified as asymptomatic. At the time of writing these three dogs were aged 4, 7 and 12 years (S6 Table). However, the 4-year-old dog had presented with questionable cerebellar signs when examined by a neurologist at the age of 2 years and 11 months, and the 7-year-old dog had possible mild ataxia of hind limbs when its gait was reviewed from video material. A skin biopsy was later obtained from the 7-year-old dog but it did not show the sweat gland vacuolization present in other affected dogs. Finally, the gait of the 12-year-old dog was evaluated as normal through video material. However, even when these three dogs were placed to the control group, the association between the disease and the variant was still highly significant (p = 3.8 x 10^-136^).

To corroborate the association results, the *ATG4D* c.1288G>A variant was screened in a cohort of 642 randomly selected dogs from 40 other breeds, including closely related breeds to the LR such as the Barbet, and the Spanish and Portuguese Water Dogs (S5 Table). The variant was not found in this sample cohort, suggesting it is not a common polymorphism, but rather has an allele distribution that is consistent with a relatively young, breed-specific disease-causing variant.

All 25 LRs homozygous for the *ATG4D* variant could be drawn into a single pedigree (S1 Fig). The 25 homozygous dogs belonged to 20 different litters. DNA samples and genotypes were obtained from altogether 22 parents and 27 unaffected full siblings of homozygous dogs. In two of the litters, one affected parent was homozygous for the variant but otherwise the sampled parents were heterozygous and unaffected. The full siblings were either heterozygous (22 out of 27) or had a wild-type genotype (5 out of 27).

### Bioinformatic analysis of the *ATG4D* missense variant

The mammalian ATG4D protein belongs to the ATG4 family of cysteine proteinases, together with ATG4A, B and C [15]. The c.1288G>A variant is located in the last exon of the canine *ATG4D* gene (Fig 3B). At the protein level, the missense variant is predicted to cause an alanine to threonine amino acid change, p.A430T. The main functional domain of the ATG4D protein, the C54 peptidase domain, is located at the center of the protein body. The amino-terminal region of the ATG4D protein is suggested to contain a PEST sequence, a caspase site and a cryptic mitochondrial target sequence, and the carboxy-terminus holds a putative Bcl-2 homology-3 (BH3) domain [16, 17]. The alanine at position 430 does not reside in any of the known domains but is centered between the peptidase domain and the BH3 motif near the carboxy-terminus (Fig 3C). The position is moderately conserved in evolution as is seen in an alignment of 41 different vertebrate species (Fig 3D). A large majority (37 out of 41) of the species has either an alanine or valine at the position, both of which are non-polar, hydrophobic amino acids, whereas four of the investigated species possess a serine residue. Alignment of all four ATG4 paralogs from human and dog revealed valine residues at the corresponding positions in ATG4A, ATG4B, and ATG4C (Fig 3E). We used the PredictSNP program to provide a consensus pathogenicity estimate from several independent prediction algorithms on the functional effect of the p.A430T change [18]. The alanine to threonine change was estimated to be neutral with 85% confidence, which would suggest that the variant does not severely disrupt the protein but may still have an effect on its function.

### Analysis of the *ATG4D* transcript

We then examined whether the *ATG4D* gene is expressed in the affected tissue and if the mutation has an effect on mRNA splicing or mRNA expression levels. We sequenced the *ATG4D* transcript using RNA samples extracted from the cerebellar cortex of two affected, two carrier and two wild-type LRs. The obtained sequences were in accordance with the reference (XM_542069.3), and the c.1288G>A variant was present in the transcripts of the two affected and two carrier dogs. We did not identify splicing defects or changes in transcript levels. Both transcript alleles were present at comparable levels in the heterozygous carrier dogs (S2 Fig). These results suggest that the canine phenotype is caused by a dysfunction at the protein level and not by any change on the transcript level.

### Histological analyses reveal altered autophagy pathway in the affected neurons

**Fig 4:**
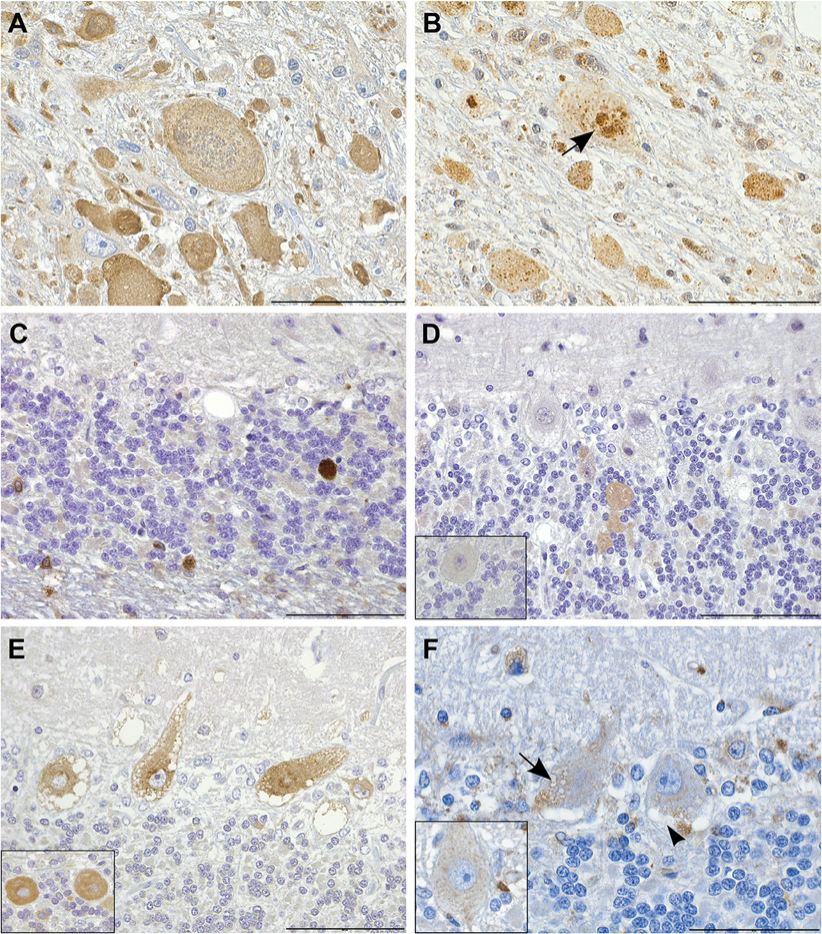
Immunohistochemistry indicates disturbed autophagic flow in neurons. (A) Axonal spheroids stain diffusely positive for LC3B. IHC LC3B, scale bar 100 μm. (B,C) The granular cores of the spheroids are positive for (B) ubiquitin (arrow) and (C) p62. IHC ubiquitin and p62, scale bars 20 and 100 μm, respectively. (D) Smooth axonal swellings in the cerebellar cortex contain ATG4D. Inset: control. IHC ATG4D, scale bar 100 μm. (E) Affected neurons show increased perinuclear granular LC3B positivity. Inset: control. IHC LC3B, scale bar 100 μm. (F) Neuronal vacuoles are partially LAMP2 positive (arrow) and partially negative (arrow head). Inset: control. IHC LAMP2, scale bar 20 μm.

We next used immunohistochemistry (IHC) to examine the nature of the neuropathological changes in more detail. For this purpose, we used antibodies produced against ATG4D, ubiquitin, the autophagosome membrane marker LC3B, the lysosome membrane marker LAMP2 and the autophagic cargo marker p62, which binds ubiquinated material destined for autophagy. The axonal spheroids showed strong diffuse immunoreactivity for LC3B (Fig 4A), and the granular core was immunoreactive for ubiquitin (Fig 4B) and p62 (Fig 4C), indicating disturbed autophagy in the neurites. Within the cerebellar granular cell layer and cerebellar white matter, the ATG4D protein was detected within the finely granular swollen axons (Fig 4D). Some vacuoles in the neuronal soma were positive for the lysosomal marker LAMP2 (Fig 4F). The ultrastructure of these single membrane bound vacuoles was consistent with distended secondary lysosomes or autolysosomes, containing digested material. Some vacuoles, however, remained unstained with all antibodies used (Fig 4F). Coarse LC3B positivity was present in the perinuclear area in several Purkinje cells, indicating induction of autophagy or blockage of the autophagic flow in the cerebellum of affected dogs (Fig 4E). Although the cause and origin of the neuronal vacuoles remains to be investigated in more detail, these results indicate alterations in the autophagy pathway in neurons of the affected dogs.

### Knockdown of zebrafish *atg4da* results in the CNS neurodegeneration

**Fig 5:**
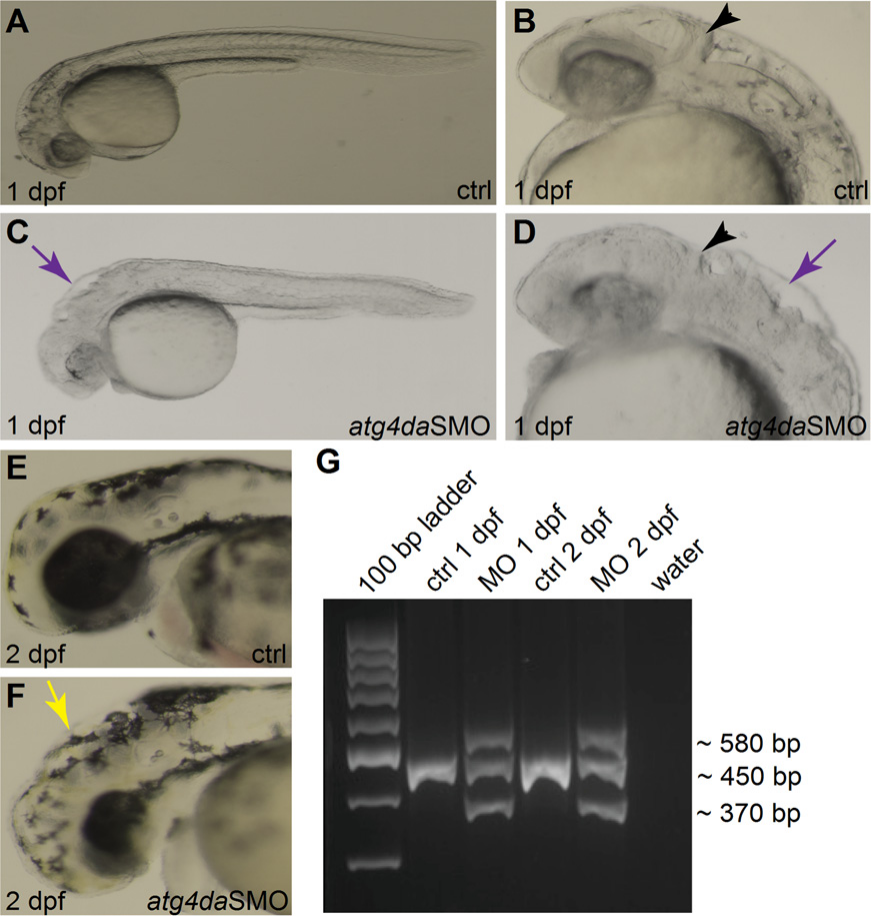
Phenotype of *atg4da* morphant zebrafish embryos. (A-F) Lateral views of control and morphant zebrafish embryos. (A,B) Control embryos appear normal at 1 dpf. (C,D) Morphant embryos show severe abnormalities in different regions of the brain at 1 dpf. Black arrowheads denote the developing cerebellum. Purple arrows indicate the hindbrain irregularities. (E) Control embryo at 2 dpf. (F) A morphant embryo at 2 dpf displaying hydrocephalus (yellow arrow) and small head and eye. (G) RT-PCR assay showing the efficiency of the *atg4da*SMO.

As the biological role of *ATG4D* is not well characterized, we decided to employ the zebrafish (*Danio rerio*) model to get insights into its neurodevelopmental role. The zebrafish model with its structural and functional similarities with mammalian organs and tissues provides an excellent platform to study gene function during development. Due to the teleost genome duplication, the mammalian *ATG4D* gene has two homologs in the zebrafish, *atg4da* and *atg4db*. The zebrafish *atg4db* gene sequence has greatly diverged from its mammalian homologs and codes for a polypeptide of less than 150 amino acids. In contrast, zebrafish *atg4da* codes for a protein of 485 residues with 54% identity to the 473 amino acid canine protein. We therefore considered zebrafish *atg4da* the functional homolog of the mammalian ATG4D protein. As a step towards understanding the functional role of *atg4da*, we carried out an oligonucleotide-based knockdown of the zebrafish *atg4da* gene by injecting a splice morpholino (*atg4da*SMO) into 1-cell staged wild-type embryos. The efficiency of the *atg4da*SMO was evaluated by RT-PCR, using primers targeting the first four exons. In comparison to a single intense band detected in standard control morpholino (stdMO) injected embryos, the *atg4da* morphant embryos showed three discrete bands; a wild-type band, a smaller band that excluded exon three (84 bp) and a larger band resulting from heteroduplex formation between the wild-type PCR product and the abnormally spliced product (Fig 5G). This indicates that the morpholino injection produced defects in proper splicing of the *atg4da* transcript.

At 26 hours post fertilization, the *atg4da* morphants displayed severe visible malformations in the developing CNS, in regions that correspond to the midbrain-hindbrain boundary, the cerebellum and the hindbrain region (Fig 5C and 5D), whereas the stdMO injected embryos appeared normal (Fig 5A and 5B). In addition, widespread neurodegeneration was present in different regions of the *atg4da* morphant brain, which could be detected as widespread dark coloration (Fig 5C and 5D). These morphological defects resulted in a small sized brain and eye, mild hydrocephalus and occasional pericardial edema in 2-day-old morphant embryos (Fig 5E and 5F). Although the defects in the developing CNS remained the same within the tested morpholino dosage range, the severity of the phenotypes was dependent on morpholino penetrance as some injected embryos showed stronger phenotypes, while the majority had milder morphological defects.

**Fig 6:**
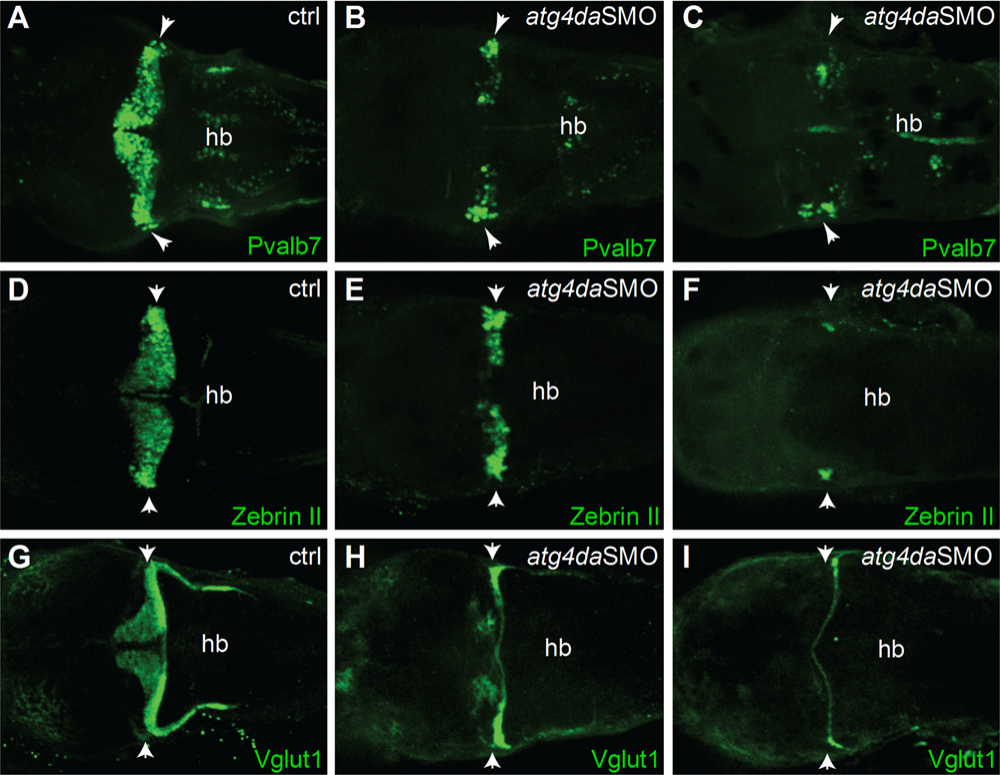
Suppression of *atg4da* in zebrafish leads to loss of cerebellar neurons. Immunostaining with (A-C) anti-Pvalb7 and (D-F) anti-zebrinII antibody show loss of cerebellar Purkinje cells in *atg4da* morphants. (A,D) Control embryos at 4.5 dpf. (B,E) Morphants with mild phenotype show partial loss of cerebellar Purkinje cells. (C,F) Morphants with strong phenotype show either total loss of Purkinje cells or presence of few differentiated neurons, which are laterally located in the cerebellum. (G-I) Labeling of cerebellar granule cells with anti-Vglut1 antibody in control and morphant embryos. (H) Mildly affected morphants show reduced expression of Vglut1 in the cerebellum. (I) Vglut1 expression is strongly reduced in embryos showing severe phenotype. White arrowheads indicate the region of cerebellum. Abbreviations: hb, hindbrain.

The ataxia phenotype in LR dogs with progressive loss of cerebellar Purkinje cells prompted us to examine the cerebellar region of *atg4da* morphants in more detail. We sought to determine whether the differentiation of the cerebellar Purkinje cells and granule cells was affected in the morphants. Immunostaining with the Purkinje cell markers parvalbumin 7 (Pvalb7) and zebrinII revealed partial loss of Purkinje cells in the cerebellum of *atg4da* morphants at 4.5 days post fertilization (dpf) (9 out of 14 morphants). In severely affected morphants, we detected either total loss of Purkinje cells or a few differentiated Purkinje cells, which were located laterally in the cerebellum (5 out of 15 morphants) (Fig 6A–6F). Immunostaining with the granule cell marker Vglut1 antibody revealed marked loss of granule cells in morphants that had a severe phenotype (4 out of 10 morphants). In morphants with a mild phenotype, the expression of Vglut1 was also reduced when compared to control embryos. These results suggest that the developmental cerebellar malformation induced by the loss of *atg4da* function could be caused by either a total loss or severe reduction of neurons in the cerebellum (Fig 6G–6I).

## Discussion

We describe a novel neurodegenerative storage disease with a recessive missense variant in the autophagy-related *ATG4D* gene. The affected dogs suffer from progressive cerebellar ataxia with a varying age of onset and disease progression. Histological findings reveal cerebellar degeneration and vacuolar changes in a variety of tissues, including neurons, secretory epithelia and cells of the mesenchymal origin. The lesions in affected dogs provide evidence of altered autophagy. Furthermore, a morpholino knockdown of the *ATG4D* zebrafish homologue revealed a neurodegenerative phenotype in the developing fish CNS.

Our genetic studies revealed a highly significant association between the canine disease and the *ATG4D* variant. The *ATG4D* gene encodes a C54 endopeptidase that is thought to function in the macroautophagy pathway [15]. Four ATG4 family members (ATG4A-D) have been identified in higher organisms [15] but their specific functional roles are poorly characterized. The yeast possesses only a single Atg4 protein, the role of which as a processor of the ubiquitin-like Atg8 protein has been extensively studied [19–21]. The yeast Atg4 cleaves Atg8 at the carboxy-terminus to reveal an evolutionary conserved glycine residue (the so called priming process), which allows the covalent attachment of the lipid phosphatidylethanolamine (PE) to the cleaved Atg8 and the subsequent attachment of the lipidated Atg8 to the forming autophagosomal membrane [20–22]. Later on, Atg4 functions as a deconjugating enzyme that delipidates Atg8, which is then recycled from the autophagosomal membranes [20, 23, 24]. The association of the Atg8-PE with the autophagosomal membrane is considered a critical step in the biogenesis of autophagosomes [23–26]. Similar to the ATG4 proteins, mammals possess several homologs of the yeast Atg8 [27]. Even though the degree of functional redundancy between the mammalian Atg4 and Atg8 homologs is not well established, differences in expression patterns [15, 28–30], functional properties [15–17, 29, 31, 32] and ATG4 substrate specificities [16, 33, 34] have been indicated. So far, no disease causing-mutations have been reported for any of the human *ATG4* genes. The effects of Atg4a and Atg4d deficiency remains to be examined in mouse models, while only mild phenotypes are seen in murine Atg4b and Atg4c knockouts, such as mild motor incoordination in Atg4b knockout mice [32, 35, 36].

To gain a better understanding of the roles of the ATG4D protein, we studied its function in a zebrafish model. The knockdown of the zebrafish Atg4da revealed a neurodegenerative phenotype with either total or partial loss of Purkinje and granule cells in the cerebellum, suggesting a functional conservation of *ATG4D* between zebrafish and dog. Although the canine and zebrafish proteins may possess species-specific functional differences, the severe phenotype of the *Atg4d* knockdown zebrafish would be in accordance with a milder effect of the missense mutation in dogs. A complete loss-of-function of the canine ATG4D would likely cause a more severe phenotype than what is seen in affected LRs. Partial function of the ATG4D protein may also explain the variable age of onset and progression in the affected dogs. Furthermore, as clinical signs could not be confirmed in three dogs homozygous for the *ATG4D* variant, the possibility of a reduced penetrance cannot be ruled out at this point.

Our results support the role of ATG4D in the autophagy-mediated neuronal homeostasis in the CNS. We show that that the *ATG4D* transcript is present in the cerebellar cortex of affected and healthy dogs. This is in accordance with publicly available RNA *in situ* hybridization data from the developing and adult murine brain [37]. The mouse *Atg4d* is widely expressed in the CNS, with especially strong expression in the adult cerebellum (http://www.informatics.jax.org/assay/MGI:4945783) [37]. Outside of the CNS, *ATG4D* gene expression has been indicated in several different tissues and organs (http://www.proteinatlas.org/ENSG00000130734-ATG4D/tissue) [15, 32]. Our results identify increased LC3B expression in the cerebellar Purkinje cells of the affected dogs, indicating an altered autophagic pathway. Autophagy is known to be critical for neuronal homeostasis, as implicated by the severe CNS phenotypes in mice that lack the autophagy pathway proteins Atg5 and Atg7 [38, 39]. The neuronal Atg5 and Atg7 knockouts present with behavioral deficits and progressive motor dysfunction, such as ataxia [38, 39]. At the histological level, neurodegeneration is present and intracytoplasmic ubiquitin-positive protein inclusions accumulate in neurons [38, 39]. Notably, the neuronal Atg5 knockout mouse show degenerative changes especially in the cerebellar Purkinje cells [38] similar to our affected dogs. Furthermore, a recent study reported corresponding histological findings in dogs with a juvenile-onset progressive cerebellar ataxia, caused by a recessive mutation in the autophagy-linked *RAB24* gene [40]. Interestingly, while the CNS changes in the affected LRs share several features with the other autophagy-impaired models, they differ by having a unique intracellular vacuolization. In addition to the neuronal findings, marked vacuolization of epithelial cells was present in several secretory organs in affected LRs, for instance in the pancreas and salivary glands. However, these changes did not appear to translate into clinical signs, such as pancreatic insufficiency. The role of autophagy proteins in extraneuronal tissue and in secretion pathways is an area of increasing interest [41], and the affected LRs provide an exciting model to further investigate the role of ATG4D in extraneuronal autophagy.

The vacuolar change in the affected dogs resembled those seen in LSDs, however, we failed to identify any specific storage material, such as glycogen, ganglioside or other glycolipids [8]. The neuronal vacuoles and the vacuoles found in phagocytic cells stained partially positive for lysosomal membrane antigens, indicating altered lysosomal homeostasis. Since the autophagy pathway and lysosomal degradation are tightly linked and have common regulatory mechanisms, it is not surprising that disturbed autophagic flow can affect lysosomal homeostasis. Cumulating evidence shows that some storage disorders considered as primary LSDs, could be caused at least in part by disturbed autophagy [13]. As the autophagosome matures and fuses with the lysosome to form the autolysosome, autophagy-related markers such as LC3B are degraded and lysosomal markers become the main membrane protein despite the autophagic origin of the vesicle [42]. Our findings of LC3B negative, partially LAMP2 positive vesicles that fuse into larger vesicles may indicate a disturbed end-phase of the autophagic degradation.

In conclusion, we have characterized a novel neurodegenerative disorder with unique histological changes that are linked to impaired autophagy. We identify a mutation in an autophagy-related protease gene, *ATG4D*, which represents a novel candidate gene for neurodegeneration. Our study establishes a novel canine model to investigate ATG4D-mediated functions in autophagy and to evaluate possible treatment options. Meanwhile, veterinary diagnostics and breeding programs benefit from a genetic test.

## Materials and Methods

### Ethics statement

All dogs used in this study were privately owned pets that were examined with the consent of their owners. The clinical and genetic experiments performed on dogs were approved by the “Cantonal Committee For Animal Experiments” (Canton of Bern; permit 23/10) and by the “Animal Ethics Committee at the State Provincial Office of Southern Finland” (permit: ESAVI/6054/04.10.03/2012). All zebrafish experiments were performed in accordance to the guidelines approved by “Stockholm North Experimental Animal committee” (Dnr N29-12).

### Animals and pedigrees

The study cohort comprised altogether 2,352 LRs and 642 dogs from 40 other breeds (S5 Table). Samples were obtained from seven LRs that were confirmed as affected by pathological examination, and from 15 LRs with corresponding clinical signs. The remaining LR samples included several relatives of affected dogs, such as parents and siblings but also unaffected dogs from the general LR population.

The disease pedigree was drawn using the GenoPro genealogy software (http://www.genopro.com/). The pedigree information was obtained from the Finnish Kennel Club’s pedigree registry KoiraNet (http://jalostus.kennelliitto.fi/), the Lagotto Pedigree database (http://lagotto.hu/database.htm) and from individual dog owners and breeders.

### Clinical examinations

Detailed clinical examination was performed on 16 of the affected dogs. Clinical examinations included general clinical and neurological examination. For the rest of the affected dogs detailed history of the dogs and their clinical signs were received by phone or email interview of the dog owners.

### Necropsy and histological examinations

Seven affected dogs underwent postmortem examination after euthanasia and skin biopsies from four live dogs were available for review. Tissue samples of internal organs, skin, central and peripheral nervous system taken during necropsy were formalin fixed and paraffin embedded for histology along with the skin biopsies. Tissue sections and skin biopsies were stained with HE, PAS and treated with diastase for glycogen digestion. Electron microscopy samples of the cerebellar cortex of one dog were fixed in 2.5% buffered glutaraldehyde, washed and contrast stained with 1% osmium tetroxide and 8% uranyl acetate in 0.69% maleic acid and embedded in epoxy resin. Ultrathin sections were mounted on copper grids, stained with Reynolds lead citrate stain and viewed in Phillips EM2085 at 80 kV. Tissue samples from four affected dogs were included in the immunohistochemical stainings. Antigens were retrieved with 0.01M citrate buffer at pH 6 and heat for 20 minutes at 99°C. The sections were stained according to the UltraVision Detection System HRP/DAB kit (Thermo Fisher Scientific Inc.) using primary antibodies against LC3B (ab48394, Abcam), p62/SQSTM1 (P0067, Sigma-Aldrich), ubiquitin (ab7780, Abcam), LAMP2 (LS-B3144, LifeSpan Biosciences Inc.) ATG4D (SAB1301447, Sigma-Aldrich), and GFAP (MCA1909, Serotec, Bio-Rad Laboratories Inc.). Tissue samples from an unaffected (homozygous wild-type for the *ATG4D* variant) 5 year-old male LR, euthanized due to heart failure, was used as control for the immunohistochemical stainings.

### DNA samples

The majority of samples were collected as whole blood, and a small proportion as buccal swabs or tissue samples. The genomic DNA was isolated from EDTA-blood using the Nucleon Bacc2 kit (GE Healthcare) or a semi-automated Chemagen extraction robot (PerkinElmer Chemagen Technologie GmbH). Tissue samples were processed by using the Chemagen robot, and buccal swabs by using the QiaAmp DNA Mini Kit (Qiagen). The concentration of DNA samples was measured by using a NanoDrop-1000 UV/Vis Spectrophotometer (Thermo Fisher Scientific Inc.). DNA samples were stored at -20°C.

### Linkage analysis and homozygosity mapping

Genotyping of three affected and four unaffected dogs was performed at the GeneSeek facility (Neogen Corporation) using Illumina’s CanineHD BeadChips containing 173,662 validated SNPs. Genotypes were stored in a BC/Gene database version 3.5 (BC/Platforms). Linkage analysis was performed using SNP chip genotypes of altogether six dogs from a single nuclear family. The family comprised the non-affected parents, two affected and two healthy siblings. The Merlin software [43] was used to perform parametric linkage analysis under a fully recessive inheritance model. The PLINK v1.07 software [44] was used to search for extended intervals of homozygosity in the three genotyped cases as described previously [45]. The final critical intervals were defined by visual inspection of all SNP chip genotypes of the three cases on canine chromosomes 11, 13, and 20 in an Excel-file.

### Gene analysis

We used the dog CanFam 3.1 assembly for all analyses. All numbering within the canine *ATG4D* gene correspond to the accessions XM_542069.3 (mRNA) and XP_542069.1 (protein).

### Whole genome sequencing of an affected dog

We prepared a fragment library with a 300 bp insert size and collected 210,168,963 Illumina HiSeq2500 paired-end reads (2 x 100 bp) corresponding to roughly 15.5x coverage. The reads were mapped to the dog reference genome using the Burrows-Wheeler Aligner (BWA) version 0.5.9-r16 [46] with default settings. This resulted in altogether 380,485,021 unique mapping reads. The Picard tools (http://sourceforge.net/projects/picard/) were used to sort the mapped reads by the sequence coordinates and to label the PCR duplicates. The Genome Analysis Tool Kit (GATK version v2.3–6) [47] was used to perform local realignment and to produce a cleaned BAM file. Variant calls were then made by using the unified genotyper module of GATK. Variant data was obtained in variant call format (version 4.0) as raw calls for all samples and sites flagged using the variant filtration module of GATK. Variant calls that failed to pass the following filters were labeled accordingly in the call set: (i) Hard to Validate MQ0 ≥ 4 & ((MQ0 / (1.0 * DP)) > 0.1); (ii) strand bias (low Quality scores) QUAL < 30.0 || (Quality by depth) QD < 5.0 || (homopolymer runs) HRun > 5 || (strand bias) SB > 0.00; (iii) SNP cluster window size 10. The snpEFF software [48] together with the CanFam 3.1 annotation was used to predict the functional effects of detected variants. We considered the following snpEFF categories of variants as non-synonymous: non_synonymous_coding, codon_deletion, codon_insertion, codon_change_plus_codon_deletion, codon_change_plus_codon_insertion, frame_shift, exon_deleted, start_gained, start_lost, stop_gained, stop_lost, splice_site_acceptor, splice_site_donor. The critical intervals on chromosomes 11, 13, and 20 contained 18,984,944 bp and 103,507 coding nucleotides, respectively. In our re-sequencing data, we had ≥ 4x coverage on 18,557,536 bp of the critical interval (97.7%) and on 98,578 (95.2%) of the coding bases.

### Sanger sequencing and TaqMan genotyping

Sanger sequencing was used to confirm the presence of the *ATG4D* candidate variant identified in the whole genome scan. Both Sanger sequencing and TaqMan genotyping were then used to genotype the variant in our full LR cohort and in dogs from 40 other breeds. Primers used in Sanger sequencing were designed by using the Primer3 program (http://frodo.wi.mit.edu/primer3/). PCR products were amplified using AmpliTaq Gold 360 Mastermix (Applied Biosystems, Life Technologies) or Biotools’ DNA Polymerase. The sequencing reactions were then performed on an ABI 3730 capillary sequencer (Applied Biosystems, Life Technologies), after treatment with exonuclease I and shrimp alkaline phosphatase. The Sanger sequence data were analyzed using Sequencher 5.1 (GeneCodes) or Variant Reporter v1.0 (Applied Biosystems, Life Technologies). The TaqMan genotyping reactions were performed using Applied Biosystems’ TaqMan chemistry and 7500 Fast Real-Time PCR instrumentation by following the manufacturer’s instructions. Primer and probe sequences used in Sanger sequencing and Taqman genotyping are listed in S7 Table.

### Tissue samples and mRNA experiments

Fresh tissue samples were collected in RNAlater solution (Ambion, Life Technologies) from two affected LRs and from four unaffected LRs that were euthanized on their owners’ request, and donated for the research. Samples were harvested immediately after euthanasia and stored in -80°C until further use. The affected dogs were 2-year-old male littermates euthanized due to progressive clinical signs. One control dog was an 11-month-old male LR that had suffered from seizures of unknown etiology. Another control dog was a 10-year old female LR that was euthanized for progressive cognitive decline. Two control dogs, a 9-year old female and a 10-year old female, were both euthanized because of a severe hip dysplasia.

Total RNA was extracted from cerebellar tissue samples by using the RNeasy Mini Kit (Qiagen), and sample concentrations were measured by using a ND-1000 UV/Vis Spectrophotometer (Thermo Fisher Scientific Inc.). The High Capacity RNA-to-cDNA Kit (Applied Biosystems, Life Technologies) was then used to reverse-transcribe equal amounts of total RNA into cDNA. The canine *ATG4D* transcript was amplified and sequenced by using six primers pairs (S7 Table) that were designed to span over exon-intron boundaries in order to control for genomic DNA contamination. The PCR amplification and sequence analysis was carried out as described in methods for Sanger sequencing. In addition, the entire ~1500 bp *ATG4D* transcript was amplified from an affected and wild-type dog using the Phusion Hot Start II High-Fidelity DNA polymerase (Thermo Fisher Scientific Inc.). The PCR products were visualized on a 2% agarose gel.

### Sequence alignment and pathogenicity prediction

A multiple sequence alignment of the *ATG4D* protein was constructed by using the Clustal Omega tool (http://www.ebi.ac.uk/Tools/msa/clustalo/). The aligned protein sequences were derived from the Entrez Protein database (http://www.ncbi.nlm.nih.gov/protein), with the exception of the zebrafish sequence, which was obtained from the Ensembl database (http://www.ensembl.org/). The aligned species were selected based on availability of sequences for the ATG4D protein at the time of writing. The possible pathogenicity of the identified amino acid change was examined by running the PredictSNP 1.0 program, which calculates a consensus value using several separate prediction programs [18].

### Zebrafish maintenance and morpholino injection

Embryos from wild-type AB strain were obtained by natural spawning and raised in Petri dishes at 28.5°C in E3 medium (5 mM NaCl, 0.17 mM KCl, 0.33 mM CaCl_2_, 0.33 mM MgSO_4_). Embryos were treated with phenylthiourea to inhibit pigmentation, and collected at different developmental stages.

The splice-morpholino, *atg4da*SMO, binding to intron1 and exon2 of *atg4da* pre-mRNA, and a stdMO were purchased from Gene Tools (S7 Table). The morpholinos were dissolved in 1x Danieu’s solution and approximately 2–3 ng/embryo were injected into 1-cell stage embryos. The specificity of the splice morpholino was evaluated by RT-PCR using primers listed in S7 Table. The PCR products were visualized on 3% agarose gel and processed for sequencing using the GenElute Gel Extraction Kit (Sigma-Aldrich). The used zebrafish *atg4da* reference sequences were ENSDART00000152289 (mRNA) and ENSDARP00000126975 (protein).

### Immunostaining of zebrafish embryos

Whole-mount immunostaining on control and morphants embryos were performed using primary antibodies anti-Pvalb7 (1:1000; mouse ascites), anti-zebrin II (1:200; hybridoma supernatant) and anti-Vglut1 (1:1000; purified antibody) as described previously [49]. Goat anti-mouse Alexa 488 (Molecular Probes, Life Technologies) was used as the secondary antibody. All images were obtained using Andor spinning disk confocal microscope at 10x magnification.

## Supporting Information

S1 Fig Disease pedigree established around affected dogs. A pedigree established around the Lagotto Romagnolo dogs homozygous for the *ATG4D* variant. The numbering of affected dogs corresponds with the numbering in S6 Table. The genotypes of sampled dogs are denoted in the pedigree. Within the affected litters, the health status of those siblings that were not sampled is not known. The dogs used in genome-wide analyses are circled and the dog used for whole genome sequencing is marked with an arrowhead.
(TIF)

S2 Fig Analysis of the *ATG4D* transcript in cerebellar tissue. (A) The *ATG4D* transcript was amplified using cDNA obtained from cerebellar cortical tissue samples of two affected, two carrier, and two wild-type dogs. Equal amounts of cDNA were used in each reaction. The band sizes and rough expression levels do not differ between affected and healthy dogs. (B) The full-length *ATG4D* transcript was amplified from cerebellar cortex of an affected and a wild-type dog, showing uniform transcript sizes and levels. (C) Chromatograms obtained from the sequencing of the *ATG4D* transcript. In the heterozygous carrier dog, both alleles are represented at roughly equal amounts. Abbreviations: wt, wild-type; het, heterozygous; mut, mutant; G, genomic DNA.
(TIF)

S1 Table Linkage analysis. (XLSX)

S2 Table Homozygosity mapping. (XLSX)

S3 Table Combined linkage and homozygosity results. (XLSX)

S4 Table Dog genomes. (XLSX)

S5 Table Sample cohort. (XLSX)

S6 Table Lagotto Romagnolo dogs homozygous for the *ATG4D* variant. (XLSX)

S7 Table Primer and probe sequences. (XLSX)

S1 Video Ataxia in an affected Lagotto Romagnolo dog. Video shows progressive ataxia in an affected female Lagotto Romagnolo dog. The progression of clinical signs was recorded at the age of 7 and 18 months. The age of disease onset in the dog was 4 months and the age at euthanasia 20 months.
(MOV)

## References

[1] Jellinger KA. Basic mechanisms of neurodegeneration: A critical update. J Cell Mol Med. 2010;14(3): 457–487. 10.1111/j.1582-4934.2010.01010.x 20070435 PMC3823450

[2] Murrow L, Debnath J. Autophagy as a stress-response and quality-control mechanism: Implications for cell injury and human disease. Annu Rev Pathol. 2013;8: 105–137. 10.1146/annurev-pathol-020712-163918 23072311 PMC3971121

[3] Amm I, Sommer T, Wolf DH. Protein quality control and elimination of protein waste: The role of the ubiquitin-proteasome system. Biochim Biophys Acta. 2014;1843(1): 182–196. 10.1016/j.bbamcr.2013.06.031 23850760

[4] Korolchuk VI, Menzies FM, Rubinsztein DC. Mechanisms of cross-talk between the ubiquitin-proteasome and autophagy-lysosome systems. FEBS Lett. 2010;584(7): 1393–1398. 10.1016/j.febslet.2009.12.047 20040365

[5] Schreiber A, Peter M. Substrate recognition in selective autophagy and the ubiquitin-proteasome system. Biochim Biophys Acta. 2014;1843(1): 163–181. 10.1016/j.bbamcr.2013.03.019 23545414

[6] Tai HC, Schuman EM. Ubiquitin, the proteasome and protein degradation in neuronal function and dysfunction. Nat Rev Neurosci. 2008;9(11): 826–838. 10.1038/nrn2499 18931696

[7] Yang Y, Coleman M, Zhang L, Zheng X, Yue Z. Autophagy in axonal and dendritic degeneration. Trends Neurosci. 2013;36(7): 418–428. 10.1016/j.tins.2013.04.001 23639383 PMC3787524

[8] Platt FM, Boland B, van der Spoel AC. The cell biology of disease: Lysosomal storage disorders: The cellular impact of lysosomal dysfunction. J Cell Biol. 2012;199(5): 723–734. 10.1083/jcb.201208152 23185029 PMC3514785

[9] Boustany RM. Lysosomal storage diseases—the horizon expands. Nat Rev Neurol. 2013;9(10): 583–598. 10.1038/nrneurol.2013.163 23938739

[10] Schultz ML, Tecedor L, Chang M, Davidson BL. Clarifying lysosomal storage diseases. Trends Neurosci. 2011;34(8): 401–410. 10.1016/j.tins.2011.05.006 21723623 PMC3153126

[11] Nishino I, Fu J, Tanji K, Yamada T, Shimojo S, Koori T, et al Primary LAMP-2 deficiency causes X-linked vacuolar cardiomyopathy and myopathy (Danon disease). Nature. 2000;406(6798): 906–910. 10972294 10.1038/35022604

[12] Wang D, Chan CC, Cherry S, Hiesinger PR. Membrane trafficking in neuronal maintenance and degeneration. Cell Mol Life Sci. 2013;70(16): 2919–2934. 10.1007/s00018-012-1201-4 23132096 PMC3722462

[13] Lieberman AP, Puertollano R, Raben N, Slaugenhaupt S, Walkley SU, Ballabio A. Autophagy in lysosomal storage disorders. Autophagy. 2012;8(5): 719–730. 10.4161/auto.19469 22647656 PMC3378416

[14] O'Brien DP, Leeb T. DNA testing in neurologic diseases. J Vet Intern Med. 2014;28(4): 1186–1198. 10.1111/jvim.12383 24962505 PMC4857950

[15] Marino G, Uria JA, Puente XS, Quesada V, Bordallo J, Lopez-Otin C. Human autophagins, a family of cysteine proteinases potentially implicated in cell degradation by autophagy. J Biol Chem. 2003;278(6): 3671–3678. 12446702 10.1074/jbc.M208247200

[16] Betin VM, Lane JD. Caspase cleavage of Atg4D stimulates GABARAP-L1 processing and triggers mitochondrial targeting and apoptosis. J Cell Sci. 2009;122(Pt 14): 2554–2566. 10.1242/jcs.046250 19549685 PMC2704886

[17] Betin VM, MacVicar TD, Parsons SF, Anstee DJ, Lane JD. A cryptic mitochondrial targeting motif in Atg4D links caspase cleavage with mitochondrial import and oxidative stress. Autophagy. 2012;8(4): 664–676. 10.4161/auto.19227 22441018 PMC3405841

[18] Bendl J, Stourac J, Salanda O, Pavelka A, Wieben ED, Zendulka J, et al PredictSNP: Robust and accurate consensus classifier for prediction of disease-related mutations. PLoS Comput Biol. 2014;10(1): e1003440 10.1371/journal.pcbi.1003440 24453961 PMC3894168

[19] Lang T, Schaeffeler E, Bernreuther D, Bredschneider M, Wolf DH, Thumm M. Aut2p and Aut7p, two novel microtubule-associated proteins are essential for delivery of autophagic vesicles to the vacuole. Embo j. 1998;17(13): 3597–3607. 9649430 10.1093/emboj/17.13.3597 PMC1170696

[20] Kirisako T, Ichimura Y, Okada H, Kabeya Y, Mizushima N, Yoshimori T, et al The reversible modification regulates the membrane-binding state of Apg8/Aut7 essential for autophagy and the cytoplasm to vacuole targeting pathway. J Cell Biol. 2000;151(2): 263–276. 11038174 10.1083/jcb.151.2.263 PMC2192639

[21] Ichimura Y, Kirisako T, Takao T, Satomi Y, Shimonishi Y, Ishihara N, et al A ubiquitin-like system mediates protein lipidation. Nature. 2000;408(6811): 488–492. 11100732 10.1038/35044114

[22] Kim J, Huang WP, Klionsky DJ. Membrane recruitment of Aut7p in the autophagy and cytoplasm to vacuole targeting pathways requires Aut1p, Aut2p, and the autophagy conjugation complex. J Cell Biol. 2001;152(1): 51–64. 11149920 10.1083/jcb.152.1.51 PMC2193654

[23] Xie Z, Nair U, Klionsky DJ. Atg8 controls phagophore expansion during autophagosome formation. Mol Biol Cell. 2008;19(8): 3290–3298. 10.1091/mbc.E07-12-1292 18508918 PMC2488302

[24] Kaufmann A, Beier V, Franquelim HG, Wollert T. Molecular mechanism of autophagic membrane-scaffold assembly and disassembly. Cell. 2014;156(3): 469–481. 10.1016/j.cell.2013.12.022 24485455

[25] Kirisako T, Baba M, Ishihara N, Miyazawa K, Ohsumi M, Yoshimori T, et al Formation process of autophagosome is traced with Apg8/Aut7p in yeast. J Cell Biol. 1999;147(2): 435–446. 10525546 10.1083/jcb.147.2.435 PMC2174223

[26] Nakatogawa H, Ichimura Y, Ohsumi Y. Atg8, a ubiquitin-like protein required for autophagosome formation, mediates membrane tethering and hemifusion. Cell. 2007;130(1): 165–178. 17632063 10.1016/j.cell.2007.05.021

[27] Shpilka T, Weidberg H, Pietrokovski S, Elazar Z. Atg8: An autophagy-related ubiquitin-like protein family. Genome Biol. 2011;12(7): 226-2011-12-7-226. 10.1186/gb-2011-12-7-226 21867568 PMC3218822

[28] Xin Y, Yu L, Chen Z, Zheng L, Fu Q, Jiang J, et al Cloning, expression patterns, and chromosome localization of three human and two mouse homologues of GABA(A) receptor-associated protein. Genomics. 2001;74(3): 408–413. 11414770 10.1006/geno.2001.6555

[29] He H, Dang Y, Dai F, Guo Z, Wu J, She X, et al Post-translational modifications of three members of the human MAP1LC3 family and detection of a novel type of modification for MAP1LC3B. J Biol Chem. 2003;278(31): 29278–29287. 12740394 10.1074/jbc.M303800200

[30] Nemos C, Mansuy V, Vernier-Magnin S, Fraichard A, Jouvenot M, Delage-Mourroux R. Expression of gec1/GABARAPL1 versus GABARAP mRNAs in human: Predominance of gec1/GABARAPL1 in the central nervous system. Brain Res Mol Brain Res. 2003;119(2): 216–219. 14625090 10.1016/j.molbrainres.2003.09.011

[31] Weidberg H, Shvets E, Shpilka T, Shimron F, Shinder V, Elazar Z. LC3 and GATE-16/GABARAP subfamilies are both essential yet act differently in autophagosome biogenesis. Embo j. 2010;29(11): 1792–1802. 10.1038/emboj.2010.74 20418806 PMC2885923

[32] Marino G, Salvador-Montoliu N, Fueyo A, Knecht E, Mizushima N, Lopez-Otin C. Tissue-specific autophagy alterations and increased tumorigenesis in mice deficient in Atg4C/autophagin-3. J Biol Chem. 2007;282(25): 18573–18583. 17442669 10.1074/jbc.M701194200

[33] Kabeya Y, Mizushima N, Yamamoto A, Oshitani-Okamoto S, Ohsumi Y, Yoshimori T. LC3, GABARAP and GATE16 localize to autophagosomal membrane depending on form-II formation. J Cell Sci. 2004;117(Pt 13): 2805–2812. 15169837 10.1242/jcs.01131

[34] Li M, Hou Y, Wang J, Chen X, Shao ZM, Yin XM. Kinetics comparisons of mammalian Atg4 homologues indicate selective preferences toward diverse Atg8 substrates. J Biol Chem. 2011;286(9): 7327–7338. 10.1074/jbc.M110.199059 21177865 PMC3044989

[35] Marino G, Fernandez AF, Cabrera S, Lundberg YW, Cabanillas R, Rodriguez F, et al Autophagy is essential for mouse sense of balance. J Clin Invest. 2010;120(7): 2331–2344. 10.1172/JCI42601 20577052 PMC2898610

[36] Read R, Savelieva K, Baker K, Hansen G, Vogel P. Histopathological and neurological features of Atg4b knockout mice. Vet Pathol. 2011;48(2): 486–494. 10.1177/0300985810375810 20634410

[37] Magdaleno S, Jensen P, Brumwell CL, Seal A, Lehman K, Asbury A, et al BGEM: An in situ hybridization database of gene expression in the embryonic and adult mouse nervous system. PLoS Biol. 2006;4(4): e86 16602821 10.1371/journal.pbio.0040086 PMC1413568

[38] Hara T, Nakamura K, Matsui M, Yamamoto A, Nakahara Y, Suzuki-Migishima R, et al Suppression of basal autophagy in neural cells causes neurodegenerative disease in mice. Nature. 2006;441(7095): 885–889. 16625204 10.1038/nature04724

[39] Komatsu M, Waguri S, Chiba T, Murata S, Iwata J, Tanida I, et al Loss of autophagy in the central nervous system causes neurodegeneration in mice. Nature. 2006;441(7095): 880–884. 16625205 10.1038/nature04723

[40] Agler C, Nielsen DM, Urkasemsin G, Singleton A, Tonomura N, Sigurdsson S, et al Canine hereditary ataxia in Old English Sheepdogs and Gordon Setters is associated with a defect in the autophagy gene encoding RAB24. PLoS Genet. 2014;10(2): e1003991 10.1371/journal.pgen.1003991 24516392 PMC3916225

[41] Deretic V, Jiang S, Dupont N. Autophagy intersections with conventional and unconventional secretion in tissue development, remodeling and inflammation. Trends Cell Biol. 2012;22(8): 397–406. 10.1016/j.tcb.2012.04.008 22677446 PMC3408825

[42] Mizushima N, Yoshimori T, Levine B. Methods in mammalian autophagy research. Cell. 2010;140(3): 313–326. 10.1016/j.cell.2010.01.028 20144757 PMC2852113

[43] Abecasis GR, Cherny SS, Cookson WO, Cardon LR. Merlin—rapid analysis of dense genetic maps using sparse gene flow trees. Nat Genet. 2002;30(1): 97–101. 11731797 10.1038/ng786

[44] Purcell S, Neale B, Todd-Brown K, Thomas L, Ferreira MAR, Bender D, et al PLINK: A tool set for whole-genome association and population-based linkage analyses. The American Journal of Human Genetics. 2007;81(3): 559–575. 17701901 10.1086/519795 PMC1950838

[45] Drogemuller C, Becker D, Brunner A, Haase B, Kircher P, Seeliger F, et al A missense mutation in the SERPINH1 gene in Dachshunds with osteogenesis imperfecta. PLoS Genet. 2009;5(7): e1000579 10.1371/journal.pgen.1000579 19629171 PMC2708911

[46] Li H, Durbin R. Fast and accurate short read alignment with burrows-wheeler transform. Bioinformatics. 2009;25(14): 1754–1760. 10.1093/bioinformatics/btp324 19451168 PMC2705234

[47] McKenna A, Hanna M, Banks E, Sivachenko A, Cibulskis K, Kernytsky A, et al The genome analysis toolkit: A MapReduce framework for analyzing next-generation DNA sequencing data. Genome Res. 2010;20(9): 1297–1303. 10.1101/gr.107524.110 20644199 PMC2928508

[48] Cingolani P, Platts A, Wang le L, Coon M, Nguyen T, Wang L, et al A program for annotating and predicting the effects of single nucleotide polymorphisms, SnpEff: SNPs in the genome of drosophila melanogaster strain w1118; iso-2; iso-3. Fly (Austin). 2012;6(2): 80–92. 10.4161/fly.19695 22728672 PMC3679285

[49] Bae YK, Kani S, Shimizu T, Tanabe K, Nojima H, Kimura Y, et al Anatomy of zebrafish cerebellum and screen for mutations affecting its development. Dev Biol. 2009;330(2): 406–426. 10.1016/j.ydbio.2009.04.013 19371731

