# Supplementary figures and images for: A Missense Change in the ATG4D Gene Links Aberrant Autophagy to a Neurodegenerative Vacuolar Storage Disease
Source: PLoS Genet. 2015 Apr 15;11(4):e1005169. doi: 10.1371/journal.pgen.1005169 (PMC4398399; doi:10.1371/journal.pgen.1005169)

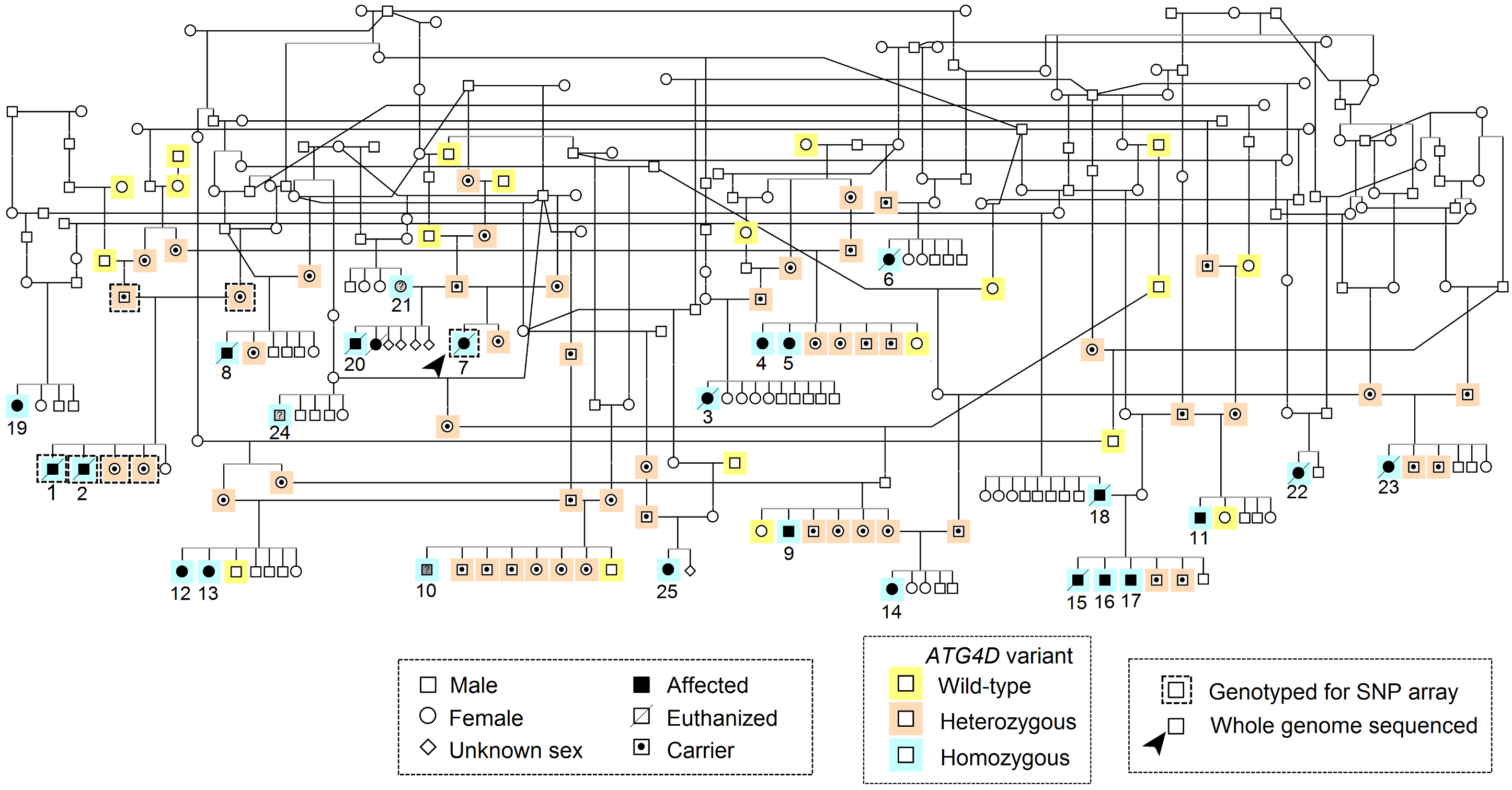

Supplement: S1 Fig — A pedigree established around the Lagotto Romagnolo dogs homozygous for the ATG4D variant. The numbering of affected dogs corresponds with the numbering in S6 Table. The genotypes of sampled dogs are denoted in the pedigree. Within the affected litters, the health status of those siblings that were not sampled is not known. The dogs used in genome-wide analyses are circled and the dog used for whole genome sequencing is marked with an arrowhead. (TIF) [file pgen.1005169.s001.tif]

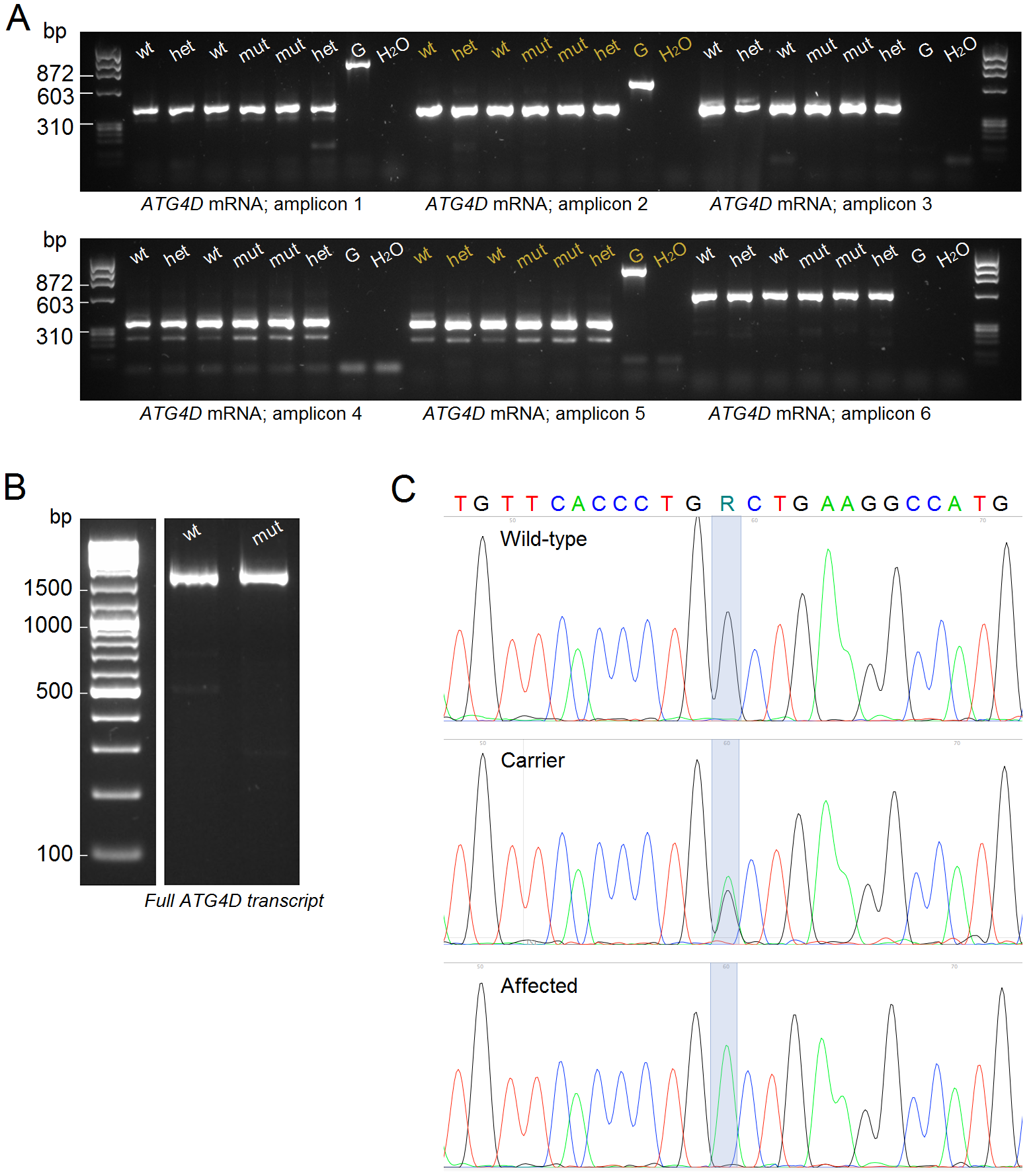

Supplement: S2 Fig — (A) The ATG4D transcript was amplified using cDNA obtained from cerebellar cortical tissue samples of two affected, two carrier, and two wild-type dogs. Equal amounts of cDNA were used in each reaction. The band sizes and rough expression levels do not differ between affected and healthy dogs. (B) The full-length ATG4D transcript was amplified from cerebellar cortex of an affected and a wild-type dog, showing uniform transcript sizes and levels. (C) Chromatograms obtained from the sequencing of the ATG4D transcript. In the heterozygous carrier dog, both alleles are represented at roughly equal amounts. Abbreviations: wt, wild-type; het, heterozygous; mut, mutant; G, genomic DNA. (TIF) [file pgen.1005169.s002.tif]
